# Assessment of the Impact of Cold Atmospheric Plasma Application on Wound Healing in Streptozotocin-Induced Diabetic Rats †

**DOI:** 10.3390/antiox15060760

**Published:** 2026-06-16

**Authors:** Emine Ersozlu, Emine Iyigun, Muhammed Kamil Turan

**Affiliations:** 1 Vocational School of Health Services, Karabük University, Karabük 78050, Turkey; 2 Faculty of Health Sciences, University of Health Sciences Türkiye, Gülhane, Ankara 06010, Turkey; emine.iyigun@sbu.edu.tr; 3 Faculty of Medicine, Karabük University, Karabük 78050, Turkey; kamilturan@karabuk.edu.tr

**Keywords:** plasma-activated water, diabetic wound healing, reactive oxygen and nitrogen species, atmospheric pressure plasma jet, Wistar albino rats, regenerative medicine, oxidative status

## Abstract

Diabetic wounds remain a major clinical challenge due to impaired healing associated with persistent inflammation, oxidative stress, and microvascular dysfunction. Plasma-based therapies have emerged as promising approaches for promoting tissue repair; however, comparative evidence regarding different plasma modalities remains limited. In this study, we evaluated and compared the effects of atmospheric pressure cold plasma (APCP) and plasma-activated water (PAW) on wound healing in a streptozotocin-induced diabetic rat model. Forty Wistar albino rats were randomly assigned to five groups: isotonic wet dressing, hydrocolloid dressing, APCP treatment, PAW application, and a non-diabetic control group. Wound healing was assessed using macroscopic evaluation, histopathological analysis, and biochemical measurements of systemic oxidative status. PAW treatment significantly accelerated wound closure during the early healing phase compared with conventional dressing methods (*p* < 0.05). Histological findings demonstrated enhanced re-epithelialization, increased collagen deposition, and improved follicular regeneration in the PAW group. Although total oxidant status (TOS) did not differ significantly among groups (*p* = 0.996), total antioxidant status (TAS) was significantly increased following PAW treatment (*p* < 0.05), indicating a more favorable systemic antioxidant profile. These findings suggest an association between improved wound healing and a more favorable systemic antioxidant profile following PAW treatment. However, because local wound-level redox parameters and molecular markers were not assessed, the contribution of redox-related mechanisms remains to be clarified. Moreover, PAW demonstrated superior therapeutic efficacy compared with direct plasma application, highlighting its potential as a non-invasive approach for diabetic wound management.

## 1. Introduction

Diabetes mellitus is a chronic metabolic disorder with a rapidly increasing global prevalence and represents a major public health concern due to its associated complications, as well as high morbidity and mortality rates [1,2]. Epidemiological data from Turkey similarly indicate a substantial rise in diabetes prevalence over recent decades, reflecting broader global trends [3,4]. Beyond its metabolic effects, diabetes significantly impairs overall health status and quality of life, primarily through the development of long-term complications affecting multiple organ systems [5,6].

Impaired wound healing is among the most severe and clinically challenging complications of diabetes. This condition arises from a complex interplay of microvascular dysfunction, persistent inflammation, and dysregulated oxidative stress, all of which contribute to delayed tissue repair [7]. In particular, disrupted redox homeostasis plays a central role in impairing early-phase wound healing, thereby prolonging the inflammatory response and delaying tissue regeneration. Diabetic foot ulcers, in particular, are associated with increased risks of infection, hospitalization, and lower extremity amputation [8,9,10,11]. Despite advances in treatment strategies, effective management of diabetic wounds remains limited, recurrence rates remain high, and the growing burden of antimicrobial resistance further complicates clinical outcomes [10,12].

In recent years, cold atmospheric plasma (CAP) has emerged as a promising therapeutic tool in biomedical research [11,13,14,15,16,17,18]. Owing to its strong antimicrobial activity and its ability to modulate cellular processes, CAP has attracted considerable attention for its potential to enhance wound healing [19,20,21]. Among plasma-based technologies, atmospheric pressure plasma jets (APPJ) have been widely investigated for applications in sterilization, tissue engineering, and direct treatment of biological tissues, particularly skin [22,23,24]. Experimental evidence suggests that APPJ systems utilizing gases such as argon, helium, or nitrogen can promote wound repair processes [25,26].

The biological effects of plasma are primarily mediated by the generation of reactive oxygen and nitrogen species (ROS/RNS), including hydrogen peroxide (H_2_O_2_), hydroxyl radicals (OH), ozone, and nitric oxide (NO) [27,28]. These species function as key regulators of cellular signaling pathways involved in inflammation, angiogenesis, and tissue regeneration. At physiological levels, ROS/RNS act as signaling mediators that support normal wound healing, whereas excessive oxidative stress disrupts cellular homeostasis and delays tissue repair [20,29]. Therefore, modulation of redox homeostasis has emerged as a critical therapeutic target in diabetic wound management.

Compared with conventional wound care strategies, plasma-based treatments offer several advantages, including targeted application and minimal invasiveness [30]. In addition to direct plasma exposure, plasma-derived solutions such as plasma-activated water (PAW) have gained increasing attention due to their ability to provide a more stable, controllable, and potentially translatable delivery of reactive species [31]. This indirect plasma approach may reduce tissue stress associated with direct plasma exposure while preserving therapeutic efficacy.

Chronic wounds continue to represent a significant global health burden, affecting millions of individuals and placing substantial strain on healthcare systems [32]. These conditions are also associated with impaired quality of life and psychosocial distress [33,34,35]. Therefore, the development of effective, mechanism-based therapeutic approaches that can accelerate early-stage healing remains a clinical priority [17,36,37]. Recent systematic reviews and meta-analyses have further highlighted the therapeutic potential of cold atmospheric plasma-based interventions in chronic wound management, supporting their growing clinical relevance and translational potential [38].

However, comparative in vivo evidence evaluating plasma-activated water and direct plasma application in diabetic wound healing remains limited. To the best of our knowledge, this study is among the few in vivo investigations that directly compare PAW, APPJ treatment, hydrocolloid dressing, and isotonic wet dressing in a streptozotocin-induced diabetic wound model.

Accordingly, the aim of the present study was to investigate and compare the effects of atmospheric pressure plasma jet (APPJ) treatment and plasma-activated water (PAW) on wound healing in a streptozotocin-induced diabetic rat model using macroscopic, histopathological, and systemic oxidative status assessments.

## 2. Materials and Methods

### 2.1. Ethical Approval and Reporting Standards

All experimental procedures were conducted in accordance with national guidelines for the care and use of laboratory animals and were approved by the Karabük University Experimental Medicine Practice and Research Center Local Ethics Committee for Animal Experiments (Protocol No: E-552112866-050.99-143801; Decision No: 2022/7/11; approved on 11 July 2022).

The study was designed, conducted, and reported in compliance with the ARRIVE (Animal Research: Reporting of In Vivo Experiments) guidelines. All personnel involved in animal handling were certified for experimental animal use.

### 2.2. Animals and Housing Conditions

Adult male Wistar albino rats were obtained from the Experimental Animal Research Center of Karabük University (Karabük, Turkey). Animals were housed under standardized laboratory conditions (12 h light/dark cycle, controlled temperature and humidity) with ad libitum access to food and water.

Animals were acclimatized prior to experimental procedures, and their health status was continuously monitored throughout the study. Animals that developed severe diabetic complications or did not meet inclusion criteria were excluded from further analysis.

### 2.3. Experimental Diabetes Model

Experimental diabetes mellitus was induced following an 18 h fasting period by a single intraperitoneal injection of streptozotocin (STZ, 60 mg/kg), prepared in cold 0.1 M citrate buffer (pH 4.5). To minimize potential circadian influences on glucose metabolism and physiological responses, STZ administration was performed at approximately 10:00 a.m. for all animals.

Blood glucose levels were measured 72 h after STZ administration, and rats with glucose levels > 250 mg/dL were considered diabetic. Blood glucose levels were monitored on days 3, 7, 10, and 13.

To prevent excessive metabolic deterioration while maintaining diabetic status, rats with severe hyperglycemia (≥600 mg/dL) received subcutaneous insulin glargine (1 IU).

### 2.4. Study Design, Randomization, and Blinding

A total of 40 male Wistar albino rats were randomly assigned to five groups (n = 8 per group):

1. Isotonic wet dressing;
2. Hydrocolloid dressing;
3. APPJ treatment;
4. Plasma-activated water (PAW);
5. Non-diabetic control.

Randomization was performed using a computer-generated sequence to ensure unbiased allocation. Outcome assessments were conducted by investigators blinded to group assignment.

Sample size was determined based on previous experimental studies evaluating wound healing in diabetic rat models.

### 2.5. Anesthesia and Surgical Procedure

All procedures were performed under general anesthesia using intraperitoneal ketamine (90 mg/kg) and xylazine (10 mg/kg).

The dorsal region was shaved and prepared under aseptic conditions. Full-thickness excisional wounds were created using a 3 mm punch biopsy on both sides of the dorsal midline. Additionally, a 10 mm longitudinal incision was created and closed with two monofilament sutures.

The study design and treatment protocol are summarized in Figure S1.

### 2.6. Treatment Protocols

Treatments were applied once daily under standardized conditions.

APPJ treatment was administered for 15 min using circular movements over the wound surface. PAW was applied by spraying directly onto the wound area.

Animals receiving plasma treatment were lightly sedated using inhalational sevoflurane (4% induction, 1–2% maintenance).

All treatment applications were initiated at approximately 10:00 a.m. each day and performed under standardized conditions to minimize potential circadian variability. Representative applications are shown in Supplementary Figures S2–S6.

### 2.7. Plasma Generation and Parameters

High-purity argon gas (99.5%) was used as the ionization source. An atmospheric pressure plasma jet (APPJ), representing a form of cold atmospheric plasma (CAP), was generated using a custom-built device developed at the Biophysics Research Laboratory of Karabük University. Argon gas was supplied at a constant flow rate of 3 L/min throughout plasma generation and treatment procedures.

The system operated at 6 kV and 30 kHz frequency, with an output power of 10 W. Surface temperature was monitored using a thermal camera (Seek XR, Seek Thermal, Santa Barbara, CA, USA), with an average temperature of approximately 40 °C. Reactive species generation was characterized using an optical emission spectrometer (ThunderOptics SMA-E, Montpellier, France; 360–920 nm). Optical emission spectroscopy (OES) and thermal imaging analyses were performed to characterize the reactive species generation and temperature profile of the atmospheric pressure plasma jet (Supplementary Figures S7 and S8).

A fixed working distance of 1 cm was maintained, and the wound area was uniformly exposed for 15 min per session. All operating parameters were kept constant throughout the study to ensure reproducibility.

#### Plasma-Activated Water (PAW) Preparation

Plasma-activated water (PAW) was generated using the same atmospheric pressure plasma jet (APPJ) system under identical operating conditions. Briefly, argon plasma was applied to 100 mL of purified water for 15 min at the same time each day. During plasma exposure, the water was continuously stirred using an electromagnetic stirrer to ensure homogeneous interaction between the plasma and the liquid phase. Immediately after generation, the freshly prepared PAW was transferred into a sterile syringe and applied directly to the wound surface by low-pressure spraying. To minimize variability associated with storage-related changes in reactive species composition, PAW was used immediately after preparation and was not stored prior to application. However, the physicochemical properties of PAW, including hydrogen peroxide, nitrite, nitrate concentrations, pH, conductivity, and storage stability, were not characterized in the present study.

For clarity, cold atmospheric plasma (CAP) is used throughout the manuscript as the general term describing plasma-based technology, whereas atmospheric pressure plasma jet (APPJ) refers specifically to the plasma source employed in the present study. Plasma-activated water (PAW) was generated using the same APPJ system and subsequently applied to the wound surface.

### 2.8. Outcome Measures and Wound Assessment

Primary Outcome:

• Wound closure rate.

Secondary Outcomes:

• Histological parameters;
• Biochemical markers.

#### 2.8.1. Wound Documentation and Image Analysis

Wound healing was documented daily (Day 0–Day 13) using a digital camera under standardized conditions. Images were captured perpendicularly from a fixed height with a scale bar for calibration.

Wound area was calculated using ImageJ software (Version 1.53, National Institutes of Health, Bethesda, MD, USA), and expressed relative to the initial wound size.

#### 2.8.2. Manual Measurements

Wound lengths were measured daily using a caliper. Measurements were performed in triplicate by the same investigator, and the mean value was recorded.

#### 2.8.3. Clinical Scoring

Wound healing was evaluated using the Bates–Jensen Wound Assessment Tool (BJWAT) by two independent blinded evaluators, including a certified wound care nurse. The mean score was used for analysis.

### 2.9. Biochemical Analysis

At the end of the experiment, blood samples were collected under deep anesthesia.

Total oxidant status (TOS) and total antioxidant status (TAS) levels were measured using commercially available assay kits (Rel Assay Diagnostics^®^, Mega Tıp Ltd., Gaziantep, Turkey).

### 2.10. Histological Analysis

Paraffin-embedded wound tissue samples were sectioned at 5 μm thickness and stained with hematoxylin–eosin (H&E) and Masson’s trichrome. Histopathological examinations were performed using a light microscope (Zeiss Axioscope 5, Carl Zeiss AG, Oberkochen, Germany). H&E staining was used to evaluate epidermal regeneration, inflammatory cell infiltration, granulation tissue formation, neovascularization, fibroblast maturation, and dermal remodeling, whereas collagen deposition and collagen organization were assessed using Masson’s trichrome staining.

Histological wound healing was evaluated using a semi-quantitative scoring system adapted from previously published studies. The scoring system included nine parameters: (a) re-epithelialization and epidermal differentiation, (b) inflammatory cell infiltration, (c) granulation tissue maturation, (d) neovascularization, (e) fibroblast maturation and orientation, (f) collagen deposition, (g) collagen organization, (h) collagen pattern, and (i) dermal maturation assessed by the presence of hair follicles.

Each parameter was assigned a score ranging from 1 to 3 according to predefined histological criteria, with higher scores indicating more advanced tissue repair and maturation. The Total Wound Healing Score (TWHS) was calculated as the sum of all individual parameter scores. Total scores of 9–13 indicated delayed wound healing, scores of 14–20 indicated moderate wound healing, and scores of 21–27 indicated advanced wound healing.

Histological sections were prepared by an experienced histologist from an independent institution who was not involved in the experimental procedures. Subsequently, stained sections were independently evaluated by two investigators blinded to treatment allocation. Representative histological images from each experimental group are presented in Supplementary Figure S11.

### 2.11. Statistical Analysis

Data distribution was assessed for normality. Non-normally distributed variables were expressed as median (minimum–maximum).

Group comparisons were performed using the Kruskal–Wallis test, followed by Dunn’s post hoc test with Bonferroni correction.

A two-tailed *p*-value < 0.05 was considered statistically significant. Statistical analyses were conducted using IBM SPSS Statistics (Version 22.0; IBM Corp., Armonk, NY, USA).

Effect sizes were also considered during the interpretation of statistically significant findings.

Variations in sample size were accounted for in the statistical analysis.

### 2.12. Mortality Monitoring

Animals were monitored daily throughout the experimental period. Mortality associated with severe diabetes mellitus and diabetic ketoacidosis occurred in several diabetic groups despite standard animal care procedures. During the first seven days, two animals each from the Wet Dressing, Hydrocolloid, and APPJ groups died. Between days 7 and 10, an additional two animals died in both the Hydrocolloid and APPJ groups. Between days 10 and 12, three animals died in the PAW group. No mortality occurred in the non-diabetic control group. All mortality events were attributed to complications associated with severe diabetes mellitus and diabetic ketoacidosis rather than treatment-related adverse effects. No treatment-associated macroscopic toxicity or procedure-related mortality was observed during the study period. Final group sizes were therefore 6 animals in the Wet Dressing group, 4 animals in the Hydrocolloid group, 4 animals in the APPJ group, 5 animals in the PAW group, and 8 animals in the Control group (Table 1).

## 3. Results

### 3.1. Diabetes-Related Findings in Rats

There were no statistically significant differences in baseline body weight among the groups. Following streptozotocin administration, diabetic rats exhibited significant weight loss and markedly elevated blood glucose levels compared with the non-diabetic control group (Table 2; *p* < 0.05), confirming the successful establishment of the experimental diabetic model.

### 3.2. Macroscopic Observation of Wound Healing

PAW treatment resulted in the most pronounced improvement in wound healing parameters compared with all other groups, particularly during the early phase of healing (day 7).

On day 7, the PAW group demonstrated the greatest reduction in incision length, punch wound length, and wound area, as confirmed by both manual measurements and ImageJ analysis (Table 3). Although all groups exhibited progressive wound reduction over time, the magnitude of improvement was significantly greater in the PAW group.

Pairwise comparisons revealed that left punch wound lengths in the PAW group were significantly shorter than those in the isotonic wet dressing, hydrocolloid, and control groups. Similarly, right punch wound lengths were significantly reduced compared with the hydrocolloid and control groups. Incision length measurements, obtained using both manual and ImageJ-based analyses, were also significantly lower in the PAW group compared with the hydrocolloid and APPJ groups.

Regarding wound area, ImageJ analysis demonstrated that left punch wound areas were significantly smaller in the PAW group compared with the wet dressing, hydrocolloid, and control groups, while right punch wound areas were significantly reduced compared with the hydrocolloid group.

By day 10, complete wound closure was observed in both the hydrocolloid and PAW groups for left punch wounds, and in the hydrocolloid, PAW, and control groups for right punch wounds. Similarly, complete incision closure was achieved in the hydrocolloid, PAW, and control groups, with statistically significant differences between groups (Table 4).

Notably, wound healing outcomes in the APPJ group were consistently inferior to those observed in the PAW and hydrocolloid groups across multiple parameters.

A very strong positive correlation was observed between manual and ImageJ-based measurements of incision length on both day 7 and day 10 (r = 0.988 and r = 1.000, respectively; *p* < 0.001), confirming the reliability of the measurement methods (Table 5).

According to the Bates–Jensen Wound Assessment Tool, the lowest (best) scores were observed in the PAW group on day 7 and among the wet dressing, PAW, and control groups on day 12 (*p* < 0.05) (Table 6). On day 7, both evaluators reported significantly better scores in the PAW group compared with the hydrocolloid and APPJ groups, whereas the APPJ group showed significantly poorer outcomes compared with the control group. Similar trends were observed on day 12.

A strong correlation between evaluators (day 7: r = 0.971; day 12: r = 0.840; *p* < 0.001) confirmed high inter-observer reliability (Table 7). Representative macroscopic images of wound healing progression in each experimental group are presented in Supplementary Figure S9.

### 3.3. Biochemical Monitoring of Wound Healing

Biochemical analysis revealed a distinct redox profile associated with PAW treatment.

On day 13, no statistically significant differences were observed in total oxidant status (TOS) levels among the groups (*p* = 0.996). In contrast, total antioxidant status (TAS) levels differed significantly between groups (*p* < 0.05), with the highest values observed in the PAW group (Table 8).

Specifically, TAS levels in the PAW group were significantly higher compared with the isotonic wet dressing and control groups.

Importantly, the higher TAS levels observed in the PAW group, in the absence of a corresponding change in TOS, indicate that PAW treatment was associated with a more favorable systemic antioxidant profile without an increase in systemic oxidant burden. Because TAS and TOS were measured only systemically and at the end of the experimental period, these findings should not be interpreted as evidence of a direct local redox mechanism. These biochemical findings are illustrated in Supplementary Figure S10.

### 3.4. Histological Monitoring of Wound Healing

Histopathological evaluation demonstrated that PAW treatment was associated with enhanced tissue regeneration compared with other groups (Table 9).

Re-epithelialization was observed in all groups; however, epithelial thickness was greatest in the PAW group, whereas the control group exhibited the lowest values. Collagen deposition was significantly lower in the control group compared with all treatment groups, while the PAW group demonstrated a higher collagen index than both the hydrocolloid and APPJ groups.

Hair follicle density was highest in the PAW group and lowest in the control group. Statistical analysis confirmed significant differences among groups in epithelial thickness, collagen index, and hair follicle density (*p* < 0.05), with the most pronounced differences observed between the PAW and control groups (Table 10).

Representative histological findings, including epithelial thickness, collagen deposition, and hair follicle density, are shown in Supplementary Figure S11A–C. Additional representative histological sections are presented in Supplementary Figure S12.

### 3.5. Integrated Interpretation of Findings

Overall, PAW treatment consistently resulted in superior wound healing outcomes compared with both conventional wound care methods and direct plasma application. This effect was observed across macroscopic, biochemical, and histological parameters, indicating a coherent and multi-level therapeutic benefit.

## 4. Discussion

The present study demonstrates that plasma-based therapies, particularly plasma-activated water (PAW), significantly enhance wound healing in a diabetic rat model compared with conventional wound care approaches. Among all treatment groups, PAW exhibited the most pronounced improvement in wound closure, especially during the early phase of healing (days 7 and 10), highlighting its potential as a promising therapeutic approach for diabetic wound management.

The accelerated healing observed in the PAW group may be associated with the biological activity of plasma-generated reactive oxygen and nitrogen species (RONS), which have been implicated in the regulation of inflammation, angiogenesis, cell proliferation, and extracellular matrix remodeling. Although diabetic wounds are characterized by excessive oxidative stress, low concentrations of reactive species may exert beneficial effects through redox-sensitive signaling pathways and hormetic biological responses. The biological effects of plasma-derived ROS/RNS are highly dose-dependent and context-dependent, with moderate levels supporting cellular signaling, angiogenesis, and tissue repair, whereas excessive levels may contribute to oxidative damage and impaired healing [14,18,39].

Previous studies support the beneficial effects of plasma-based therapies on wound repair. Cheng et al. demonstrated that argon plasma treatment increased hydrogen peroxide, nitrite, and nitrate levels, thereby promoting TGF-β expression, collagen synthesis, and re-epithelialization during early wound healing [30]. Similarly, Bogdanov et al. reported accelerated wound healing following plasma treatment in both diabetic and non-diabetic models [40]. Furthermore, Bakhtiyari-Ramezani et al. showed that plasma-activated media may induce favorable biological responses through modulation of intracellular reactive species [41].

However, not all studies have reported consistent findings. Duchesne et al. observed no significant difference between APPJ and plasma-activated medium applications [42]. Such discrepancies may be attributed to differences in plasma generation systems, gas composition, power settings, exposure duration, and treatment protocols. These observations highlight the importance of optimizing plasma conditions to achieve reproducible and clinically relevant outcomes.

A possible explanation for the superior performance of PAW compared with direct APPJ treatment may be related to differences in the delivery and persistence of plasma-generated reactive species. Plasma-activated water serves as a liquid reservoir for relatively longer-lived reactive oxygen and nitrogen species, allowing a more homogeneous and sustained exposure of the wound surface [43]. In contrast, direct plasma treatment primarily delivers short-lived reactive species whose biological effects are highly dependent on local treatment conditions, exposure distance, and tissue characteristics. Furthermore, repeated direct exposure may result in localized fluctuations in reactive species concentrations, whereas PAW may provide a more stable and evenly distributed biochemical environment. Although these mechanisms were not directly investigated in the present study, they may partly explain the enhanced wound healing outcomes observed in the PAW group and should be explored in future mechanistic studies.

This interpretation is further supported by recent experimental evidence demonstrating that topical PAW application can accelerate wound healing while simultaneously reducing bacterial burden in vivo, highlighting its potential as a clinically adaptable plasma-based therapy [31].

In the present study, conventional wound care approaches such as hydrocolloid dressing demonstrated slower healing during the early phase of wound repair. Although isotonic wet dressing provided some benefit, it appeared less effective than plasma-based interventions in sustaining tissue regeneration. These findings suggest that plasma-derived treatments may offer advantages in challenging wound-healing environments such as diabetes.

The strong agreement between macroscopic measurements and Bates–Jensen Wound Assessment Tool scores, together with high inter-observer correlation, supports the reliability and consistency of the assessment methods used in this study. This methodological agreement strengthens confidence in the comparative findings observed among treatment groups.

Biochemical analyses revealed that plasma-treated groups exhibited increased total antioxidant status (TAS), whereas total oxidant status (TOS) remained unchanged. These findings indicate an association between plasma treatment and a more favorable systemic antioxidant profile. However, because TAS and TOS are broad systemic markers, they do not provide direct evidence regarding local wound redox status or specific antioxidant pathways. Consequently, the observed changes should be interpreted as systemic associations rather than proof of a causal redox mechanism. Nevertheless, these findings are consistent with previous studies reporting beneficial effects of plasma-based therapies on oxidative stress-related parameters in diabetic wound models [30,44].

The superior healing observed in the PAW group may be related to differences in the delivery of plasma-generated reactive species compared with direct plasma exposure. One possible explanation is that PAW may provide a more homogeneous or sustained exposure to long-lived reactive species. However, because the physicochemical characteristics of PAW, including hydrogen peroxide, nitrite, nitrate concentrations, pH, conductivity, and storage stability, were not evaluated in the present study, this interpretation remains speculative and requires further investigation.

Histological findings further support the observed therapeutic benefits, demonstrating increased epidermal thickness, enhanced collagen deposition, and greater hair follicle density in plasma-treated groups. These findings are consistent with previous reports indicating that plasma-based therapies may promote keratinocyte proliferation, extracellular matrix remodeling, and tissue regeneration [39,45,46,47,48,49].

Taken together, the findings of this study demonstrate that PAW was associated with improved wound healing outcomes compared with conventional wound care and direct plasma application. Although redox-related mechanisms may contribute to these effects, the present study was not designed to establish mechanistic causality. Additional molecular and tissue-level investigations are required to clarify the biological pathways involved.

To the best of our knowledge, this study is among the few in vivo investigations that directly compare PAW, APPJ treatment, hydrocolloid dressing, and isotonic wet dressing in a streptozotocin-induced diabetic wound model.

Despite these promising findings, several limitations should be acknowledged. First, the study was conducted in an experimental animal model, and translation of these findings to clinical settings requires further validation. Second, local wound-level redox parameters, oxidative damage markers, antioxidant enzyme activities, and molecular signaling pathways were not evaluated. Therefore, mechanistic interpretations regarding redox-related effects remain preliminary. Third, the physicochemical characteristics of plasma-activated water, including hydrogen peroxide, nitrite, nitrate concentrations, pH, conductivity, and storage stability, were not assessed [43]. Consequently, interpretations regarding the mechanisms underlying the observed therapeutic effects should be considered cautiously. Fourth, sham plasma and non-activated water control groups were not included in the study, which limits the ability to distinguish plasma-specific effects from other potential treatment-related factors. Fifth, mortality associated with the diabetic model resulted in unequal final group sizes among treatment groups and may have reduced statistical power. In addition, attrition bias cannot be completely excluded, as surviving animals may not have been fully representative of the initially randomized populations. Mortality was attributed to complications associated with severe diabetes mellitus and diabetic ketoacidosis rather than treatment-related adverse effects. Sixth, blood samples were collected under deep anesthesia, which may have influenced systemic oxidative stress measurements. Although all animals were subjected to the same anesthesia and sampling protocol, a potential effect of anesthetic agents on TAS and TOS values cannot be completely excluded.

In addition, wound healing in rodents differs from human wound healing because rodents possess the panniculus carnosus muscle, which contributes substantially to wound contraction. In contrast, human diabetic ulcers heal predominantly through granulation tissue formation and re-epithelialization. Furthermore, the wounds evaluated in the present study were acute, experimentally induced wounds created under controlled laboratory conditions, whereas human diabetic ulcers are typically chronic wounds characterized by prolonged inflammation, complex comorbidities, and mature microbial biofilms. Therefore, caution should be exercised when extrapolating these findings directly to clinical settings.

Future studies should focus on characterizing the physicochemical properties of PAW, evaluating local wound-level redox markers, and investigating the molecular pathways involved in plasma-mediated wound repair. Integration of molecular, biochemical, and imaging approaches may provide a more comprehensive understanding of plasma–tissue interactions and facilitate clinical translation.

Overall, these findings suggest that PAW may represent a promising therapeutic approach for enhancing wound healing in diabetic conditions. Further mechanistic and translational studies are needed to clarify the biological basis of these effects and their potential clinical relevance.

## 5. Conclusions

In conclusion, the findings of this study demonstrate that plasma-activated water (PAW), generated using an atmospheric pressure plasma jet (APPJ), significantly enhanced wound healing in a streptozotocin-induced diabetic rat model and showed superior therapeutic performance compared with conventional wound care approaches. PAW treatment accelerated wound closure, particularly during the early phase of healing, and was associated with favorable histological outcomes, including enhanced re-epithelialization, collagen deposition, and tissue regeneration.

In addition, PAW treatment was associated with increased total antioxidant status without a corresponding increase in total oxidant status, indicating a more favorable systemic antioxidant profile. However, because local wound-level redox parameters, oxidative damage markers, antioxidant enzyme activities, and molecular signaling pathways were not assessed, the present findings do not establish a direct causal relationship between systemic oxidative status and wound healing outcomes.

Taken together, the results suggest that PAW may represent a promising and minimally invasive therapeutic approach for diabetic wound management. Although redox-related mechanisms may contribute to the observed therapeutic effects, the present study was not designed to establish mechanistic causality. Therefore, further studies incorporating local biochemical, molecular, and tissue-level analyses are required to clarify the biological pathways involved.

Future investigations should also focus on characterizing the physicochemical properties of PAW, including reactive species composition, pH, conductivity, and storage stability, as well as evaluating its therapeutic efficacy in clinically relevant settings. Such studies may provide a more comprehensive understanding of plasma-mediated wound repair and support the translational potential of PAW-based therapies.

## Figures and Tables

**Table 1:** Animal Mortality and Group Sizes Throughout the Experimental Period.

Group	Initial n	Day 7	Day 10	Final n (Day 12)
Wet Dressing	8	6	6	6
Hydrocolloid	8	6	4	4
APPJ	8	6	4	4
PAW	8	8	8	5
Non-diabetic Control	8	8	8	8

All mortality events in diabetic groups were attributed to complications associated with severe diabetes mellitus and diabetic ketoacidosis. No treatment-related mortality was observed.

**Table 2:** Baseline and longitudinal comparison of body weight and blood glucose levels among experimental groups.

	Wet Dressing (n = 8)	Hydrocolloid (n = 8)	APPJ (n = 8)	PAW (n = 8)	Control (n = 8)	*p*-Value
**Weight (g)**						
Beginning	247 (170–300)	240 (200–280)	231 (180–260)	237 (210–300)	251 (230–280)	*p* ˃ 0.05
Day 0	255 (170–300)	237 (200–280)	230 (180–260)	225 (210–300)	247 (230–280)	*p* < 0.04
Day 7	183 (142–255)	200 (164–255)	204 (155–220)	200 (145–265)	260 (239–291)	*p* < 0.002
Day 10	194 (150–255)	209 (205–235)	214 (168–225)	191 (160–262)	262 (248–289)	*p* < 0.008
Day 13	185 (119–233)	199 (169–231)	207 (185–215)	190 (175–243)	263 (202–291)	*p* < 0.013
**Glucose (mg/dL)**						
Day 0	517 (467–600)	600 (580–600)	600 (505–600)	565 (507–600)	110 (96–119)	*p* ˂ 0.000
Day 7	517 (228–600)	512 (373–600)	600 (489–600)	512 (450–600)	99 (88–115)	*p* ˂ 0.001
Day 10	412 (160–600)	426 (223–600)	410 (127–600)	475 (182–600)	98 (91–105)	*p* ˂ 0.002
Day 13	291 (98–600)	335 (135–600)	488 (121–551)	476 (236–600)	100 (92–108)	*p* ˂ 0.006

Data are presented as median (minimum–maximum). Sample sizes vary due to exclusion of animals during the experimental period.

**Table 3:** Comparison of wound length, incision length, and wound area measured by manual and ImageJ analysis on day 7 across experimental groups.

	Wet Dressing (n = 6)	Hydrocolloid (n = 6)	APPJ (n = 6)	PAW (n = 8)	Control (n = 8)	*p*-Value
**Wound length (mm)**						
**Left punch** **(Manual)**	3.2 (2.3–3.9)	3.2 (1.8–4.1)	2.9 (2.2–3.9)	1.9 (1.6–2.3)	2.9 (2.4–3.4)	0.004 *
**Right punch** **(Manual)**	2.8 (2.3–3.0)	3.2 (2.2–4.1)	2.7 (2.4–3.2)	1.6 (1.1–2.6)	2.9 (2.1–4.1)	0.002 **
**Incision** **(Manual)**	6.5 (4.9–8.8)	9.3 (8.1–14.2)	8.8 (8.0–10.2)	5.2 (0.0–7.0)	8.4 (4.0–10.4)	0.001 ***
**Incision** **(ImageJ)**	6.2 (4.7–9.3)	9.3 (7.4–14.4)	8.6 (7.9–10.3)	5.3 (0.0–6.9)	8.5 (4.1–10.3)	0.001 †
**Wound area (mm^2^)**						
**Left punch** **(ImageJ)**	0.306 (0.021–0.032)	0.0306 (0.027–0.033)	0.0275 (0.023–0.032)	0.0211 (0.015–0.025)	0.0301 (0.020–0.031)	0.004 ‡
**Right punch** **(ImageJ)**	0.0303 (0.023–0.033)	0.0321 (0.024–0.042)	0.029 (0.023–0.033)	0.0196 (0.0187–0.03)	0.029 (0.021–0.041)	0.004 §

Data are presented as median (minimum–maximum). Sample sizes vary due to exclusion of animals during the experimental period. * The difference between the PAW group and the Wet Dressing, Hydrocolloid, and Control groups is statistically significant. ** The difference between the PAW group and the Hydrocolloid and Control groups is statistically significant. *** The difference between the PAW group and the Hydrocolloid and APPJ groups is statistically significant. † The difference between the PAW group and the Hydrocolloid and APPJ groups is statistically significant. ‡ The difference between the PAW group and the Wet Dressing, Hydrocolloid, and Control groups is statistically significant. § The difference between the PAW group and the Hydrocolloid group is statistically significant.

**Table 4:** Comparison of wound healing parameters on day 10 among experimental groups.

	Wet Dressing (n = 6)	Hydrocolloid (n = 4)	APPJ (n = 4)	PAW (n = 8)	Control (n = 8)	*p*-Value
**Wound length (mm)**						
Left punch (manual)	1.3 (0.0–2.4)	0.0 (0.0–1.7)	2.3 (2.0–2.4)	0.00 (0.0–1.3)	1.4 (0.0–1.8)	0.009 *
Right punch (manual)	0.6 (0.0–2.1)	0.0 (0.0–1.6)	2.4 (2.3–2.9)	0.0 (0.0–1.7)	0.0 (0.0–1.8)	0.014 **
Incision (manual)	1.6 (0.0–3.9)	0.0 (0.0–5.5)	6.3 (6.0–9.8)	0.0 (0.0–4.0)	0.0 (0.0–3.3)	0.009 ***
İncision (ImageJ)	1.6 (0.0–3.9)	0.0 (0.0–5.5)	6.3 (6.0–9.8)	0.0 (0.0–4.0)	0.0 (0.0–3.3)	0.009 †
**Wound area (mm^2^)**						
Left punch (ImageJ)	0.1230 (0.00–0.034)	0.0000 (0.00–0.010)	0.0217 (0.020–0.026)	0.0000 (0.00–0.012)	0.0122 (0.00–0.037)	0.014 ‡
Right punch (ImageJ)	0.0069 (0.00–0.021)	0.0000 (0.00–0.00)	0.0243 (0.023–0.025)	0.0000 (0.00–0.019)	0.0000 (0.00–0.022)	0.007 §

Data are presented as median (minimum–maximum). Sample sizes vary due to exclusion of animals during the experimental period. * The difference between the APPJ group and the Hydrocolloid and PAW groups is statistically significant. ** The difference between the APPJ group and the Hydrocolloid, PAW, and Control groups is statistically significant. *** The difference between the APPJ group and the PAW and Control groups is statistically significant. † The difference between the APPJ group and the PAW and Control groups is statistically significant. ‡ The difference between the APPJ group and the PAW group is statistically significant. § The difference between the APPJ group and the Hydrocolloid and PAW groups is statistically significant.

**Table 5:** Correlation between manual and ImageJ-based wound length measurements.

	Manual Day 7	ImageJ Day 7	Manual Day 10	ImageJ Day 10
**Manual** **Day 7**		r = 0.988 *p* < 0.001		
**ImageJ** **Day 7**	r = 0.988 *p* < 0.001			
**Manual** **Day 10**				r = 1.000 *p* < 0.001
**ImageJ** **Day 10**			r = 1.000 *p* < 0.001	

Data are presented as median (minimum–maximum). Sample sizes vary due to exclusion of animals during the experimental period. Spearman correlation analysis was used. *p* < 0.001.

**Table 6:** Comparison of Bates–Jensen Wound Assessment Tool scores among experimental groups.

		Wet Dressing (n = 6)	Hydrocolloid (n = 6)	APPJ (n = 6)	PAW (n = 8)	Control (n = 8)	*p*-Value
Day 7	First Researcher	15 (13–21)	19 (19–21)	23 (21–25)	11 (10–15)	11 (10–15)	0.000 *
	Second Researcher	16 (14–20)	19 (19–20)	23 (21–25)	10 (10–15)	14 (13–15)	0.000 **
		**Wet Dressing (n = 6)**	**Hydrocolloid (n = 4)**	**APPJ** **(n = 4)**	**PAW** **(n = 5)**	**Control** **(n = 8)**	***p*****-Value**
Day 12	First Researcher	9 (9–12)	18 (11–19)	19 (19–23)	9 (9–10)	9 (9–10)	0.001 ***
	Second Researcher	9 (9–11)	17 (15–20)	19 (19–23)	9 (9–10)	9 (9–10)	0.000 †

Data are presented as median (minimum–maximum). Sample sizes vary due to exclusion of animals during the experimental period. * There is a statistically significant difference between the PAW group and the Hydrocolloid and APPJ groups, as well as between the APPJ group and the Control group. ** There is a statistically significant difference between the PAW group and the Hydrocolloid and APPJ groups, as well as between the APPJ group and the Control group. *** There is a statistically significant difference between the PAW group and the Hydrocolloid and APPJ groups, as well as between the APPJ group and the Control group. † There is a statistically significant difference between the PAW group and the Hydrocolloid and APPJ groups, as well as between the APPJ group and the Wet Dressing and Control groups.

**Table 7:** Correlation between evaluators in Bates–Jensen Wound Assessment Tool scores.

	First Researcher Day 7	Second Researcher Day 7	First Researcher Day 12	Second Researcher Day 12
**First Researcher** **Day 7**		r = 0.971 *p *< 0.001		
**Second Researcher** **Day 7**	r = 0.971 *p *< 0.001			
**First Researcher** **Day 12**				r = 0.840 *p *< 0.001
**Second Researcher** **Day 12**			r = 0.840 *p *< 0.001	

Data are presented as median (minimum–maximum). Sample sizes vary due to exclusion of animals during the experimental period. Spearman correlation analysis was used.

**Table 8:** Comparison of total antioxidant status (TAS) and total oxidant status (TOS) among experimental groups.

	Wet Dressing (n = 6)	Hydrocolloid (n = 4)	APPJ (n = 4)	PAW (n = 5)	Control (n = 8)	*p*-Value
**TAS**	1.33 (0.96–1.47)	1.21 (0.53–1.75)	1.47 (1.37–1.94)	1.54 (1.48–2.59)	1.33 (0.77–1.96)	0.033
**TOS**	0.45 (0.35–0.62)	0.45 (0.26–0.63)	0.46 (0.30–0.52)	0.44 (0.35–0.57)	0.44 (0.33–0.52)	0.996

Data are presented as median (minimum–maximum). Sample sizes vary due to exclusion of animals during the experimental period.

**Table 9:** Comparison of total wound healing scores among experimental groups.

Groups	Parameters										
	a	b	c	d	e	f	g	h	i	TWHS	Comment
Wet Dressing	13	15	16	11	13	13	16	17	18	22	Advanced
Hydrocolloid	12	15	17	9	12	10	13	15	14	19.5	Moderate
APPJ	17	16	15	11	9	13	13	12	14	20	Moderate
PAW	16	15	16	15	17	15	16	16	18	24	Advanced
Control	8	8	6	8	8	6	7	8	10	11.5	Delayed

Data are presented as median (minimum–maximum). Sample sizes vary due to exclusion of animals during the experimental period. a = Re-epithelialization, b = Inflammatory cell infiltration, c = Granulation tissue maturation, d = Neovascularization, e = Fibroblast maturation, f = Collagen deposition, g = Collagen organization, h = Collagen pattern, i = Dermal maturation. TWHS: Total wound healing scores.

**Table 10:** Comparison of histological parameters (epidermal thickness, collagen index, and hair follicle density) among experimental groups.

	Wet Dressing (n = 6)	Hydrocolloid (n = 4)	APPJ (n = 4)	PAW (n = 5)	Control (n = 8)	*p*-Value
**Epidermal thickness**	115 (112–133)	99 (82–115)	98 (83–125)	136 (125–137)	74 (70–79)	0.001
**Collagen index**	36 (33–39)	28 (22–31)	34 (24–37)	44 (35–46)	20 (18–20)	0.001
**Hair follicles**	70 (68–72)	66 (63–68)	67 (65–70)	76 (73–78)	52 (50–57)	0.001

Data are presented as median (minimum–maximum). Sample sizes vary due to exclusion of animals during the experimental period.

## Data Availability

The raw data supporting the conclusions of this article will be made available by the authors on request.

## Supplementary Materials

The following supporting information can be downloaded at: https://www.mdpi.com/article/10.3390/antiox15060760/s1, Supplementary Figure S1: Experimental design and treatment workflow of the diabetic wound model. Supplementary Figures S2–S6: Wound creation and suturing procedure. Supplementary Figure S7: Optical emission spectrum of the plasma jet measured using an optical spectrometer. Supplementary Figure S8: Measurement of plasma jet temperature using a thermal camera. Supplementary Figure S9: Representative images of wound healing progression during the experimental period. Supplementary Figure S10: Comparison of total antioxidant status (TAS) and total oxidant status (TOS) levels among experimental groups. Supplementary Figure S11: Quantitative histological analysis of epithelial thickness, collagen deposition, and hair follicle density. Supplementary Figure S12: Representative hematoxylin and eosin (H&E)-stained histological sections.
