# Supplementary material for: Assessment of the Impact of Cold Atmospheric Plasma Application on Wound Healing in Streptozotocin-Induced Diabetic Rats
Source: Antioxidants (Basel). 2026 Jun 16;15(6):760. doi: 10.3390/antiox15060760 (PMC13295428; doi:10.3390/antiox15060760)
Supplement: Supplementary file 1 [file antioxidants-15-00760-s001.zip › antioxidants-4351978-supplementary.pdf]

Supplementary Figures

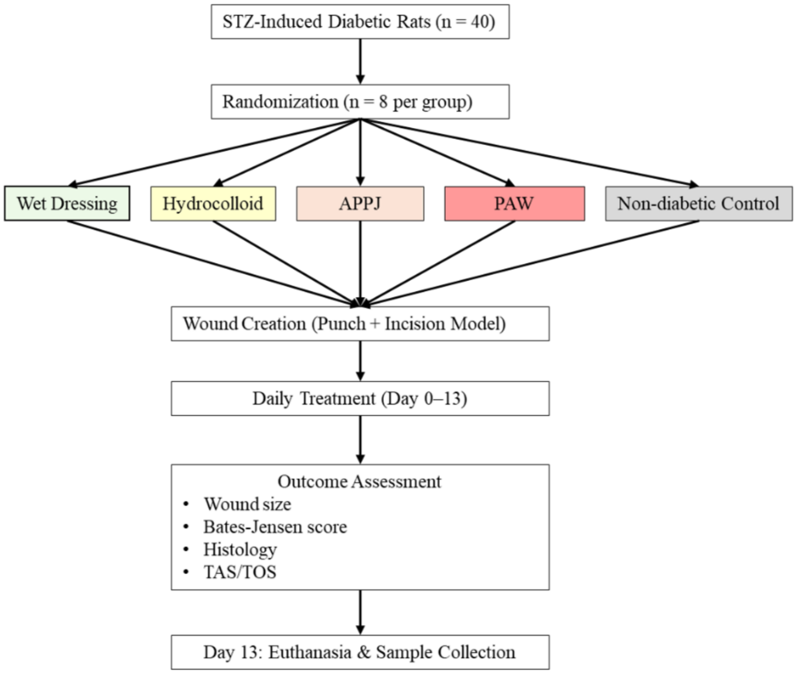

Figure S1. Experimental design and treatment workflow of the diabetic wound model.
Experimental design and treatment workflow of the diabetic wound model.Rats were randomly allocated into five groups (n = 8 per group): wet dressing, hydrocolloid dressing, atmospheric pressure plasma jet (APPJ), plasma-activated water (PAW), and control. Diabetes was induced using streptozotocin (STZ), followed by standardized wound creation. Treatments were applied daily for 13 days. Wound healing was assessed using macroscopic measurements, the Bates-Jensen Wound Assessment Tool, histological analysis, and biochemical parameters including total antioxidant status (TAS) and total oxidant status (TOS).

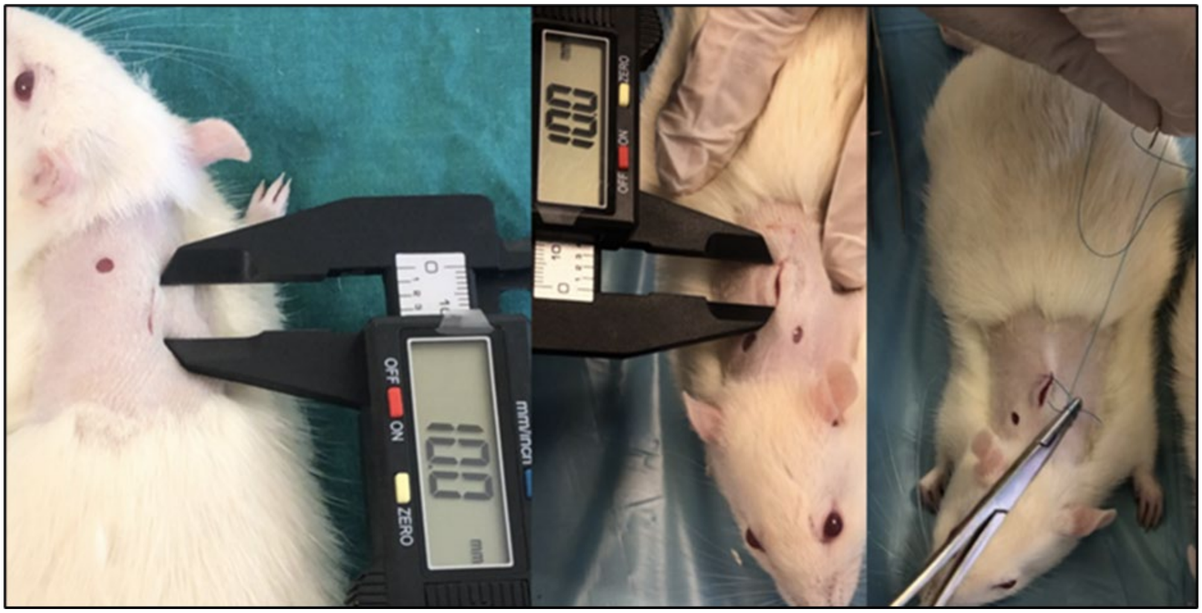

Figure S2. Wound creation and suturing procedure.

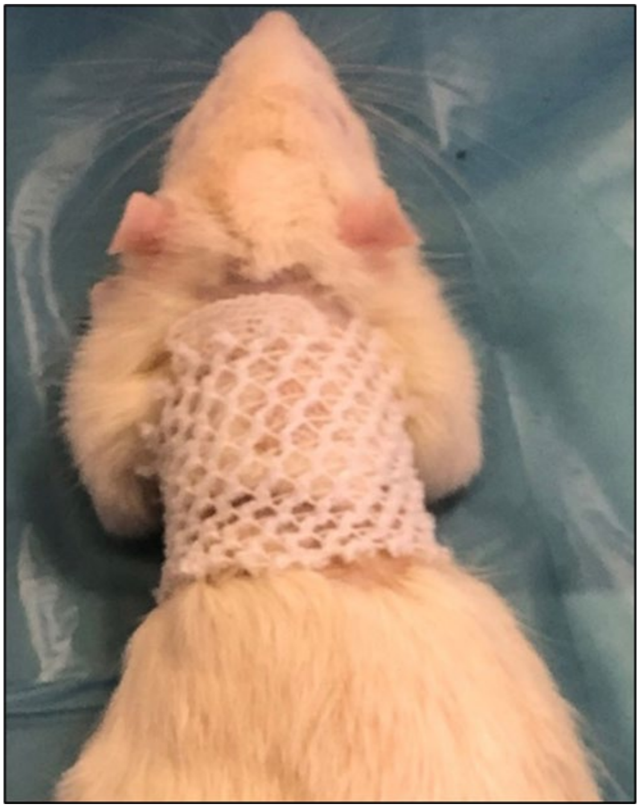

Figure S3. Application of isotonic wet dressing.

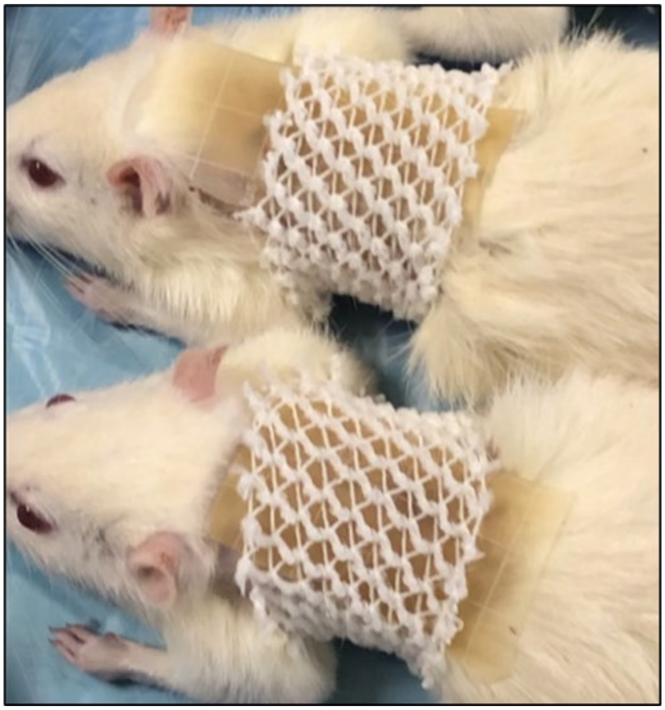

Figure S4. Application of hydrocolloid dressing.

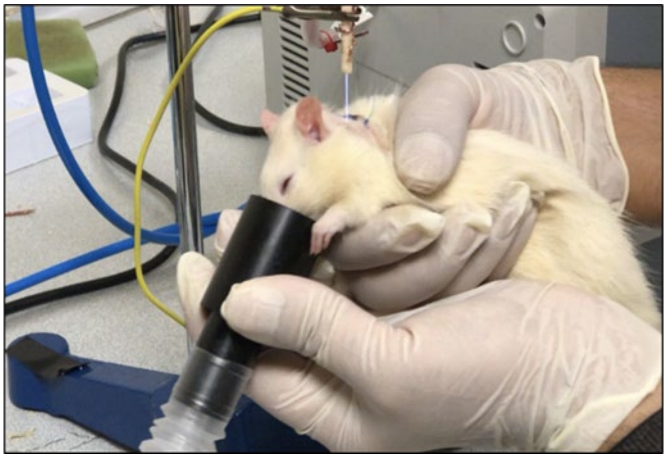

Figure S5. Application of atmospheric pressure plasma jet (APPJ).

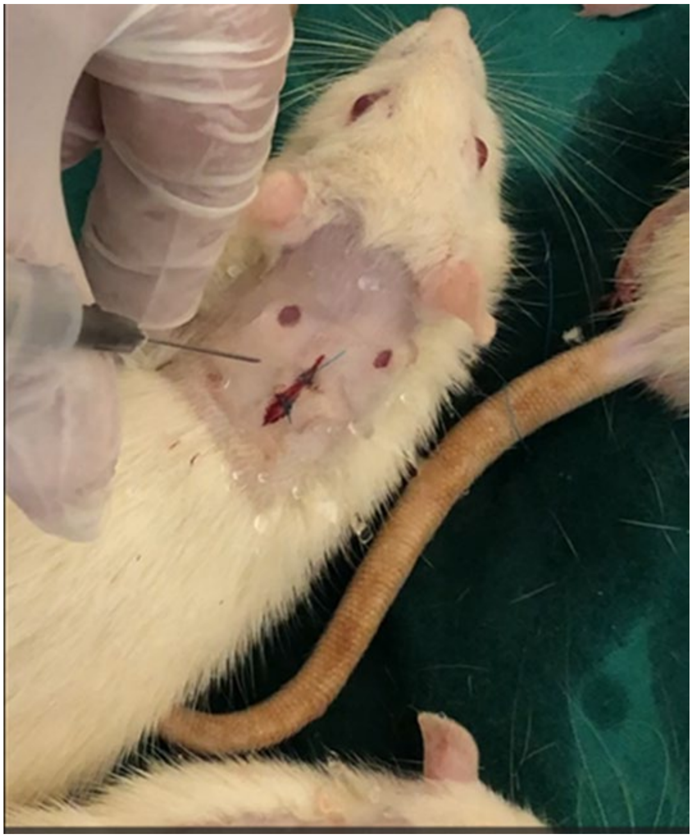

Figure S6. Application of plasma-activated water (PAW).

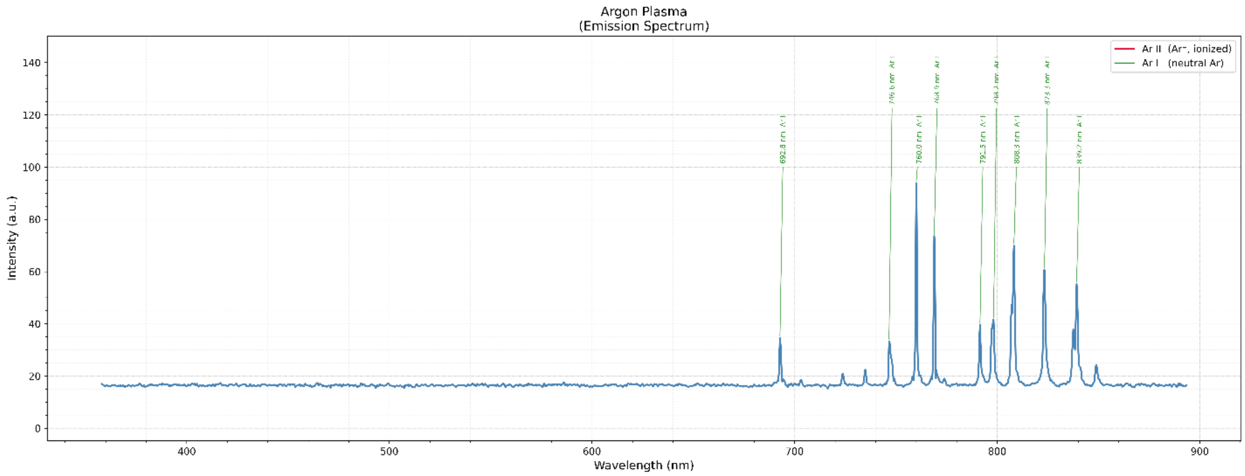

Figure S7. Optical emission spectroscopy (OES) profile of the atmospheric pressure plasma jet showing the characteristic emission peaks of reactive species.

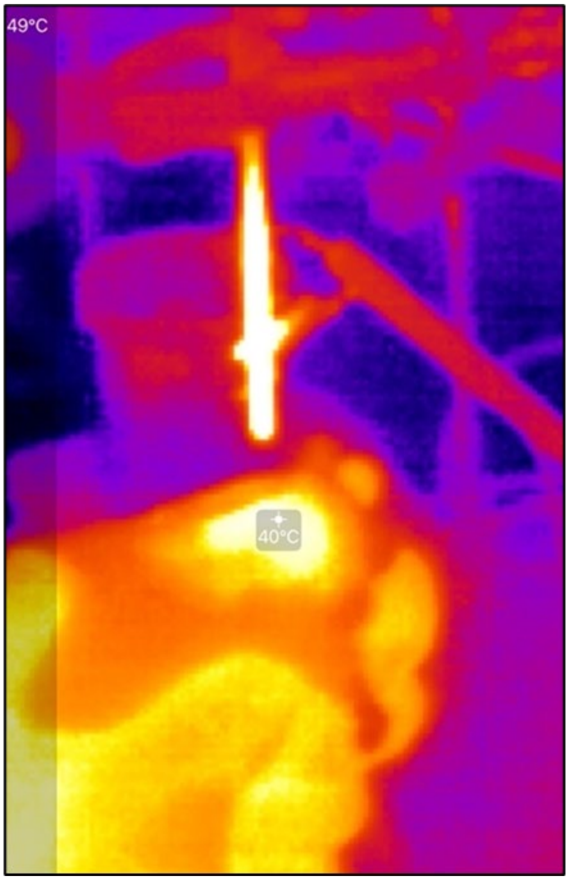

Figure S8. Measurement of plasma jet temperature using a thermal camera.

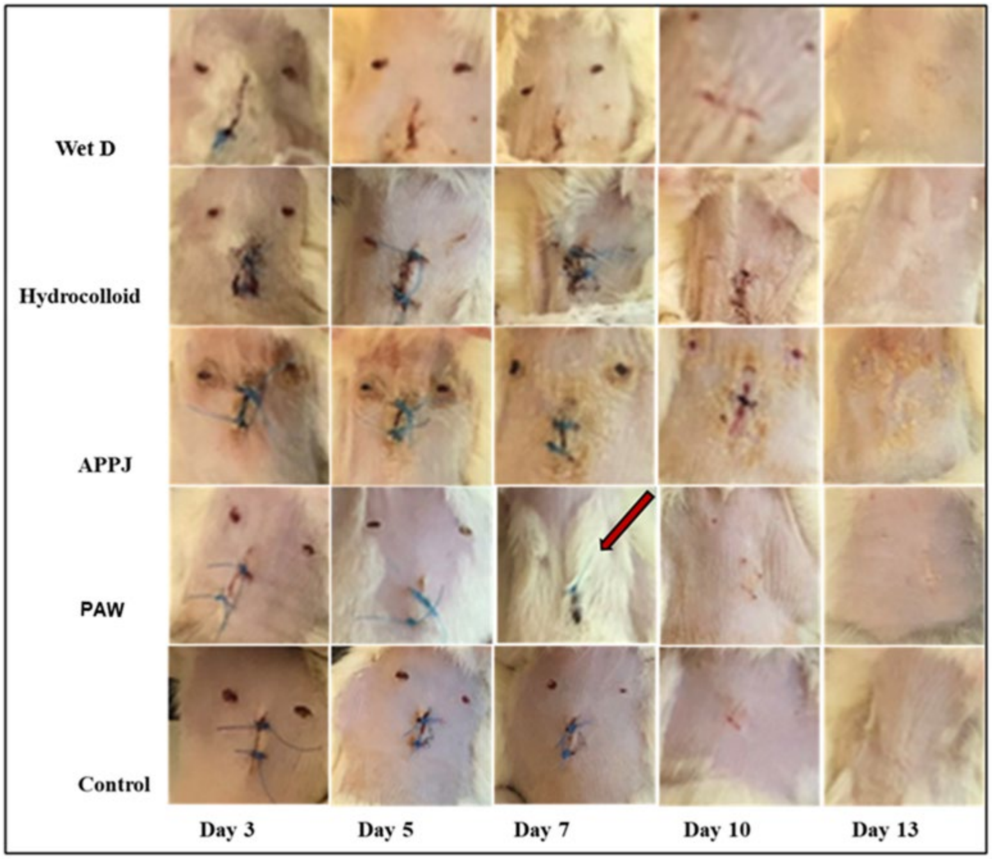

Figure S9. Representative images of wound healing progression during the experimental period.

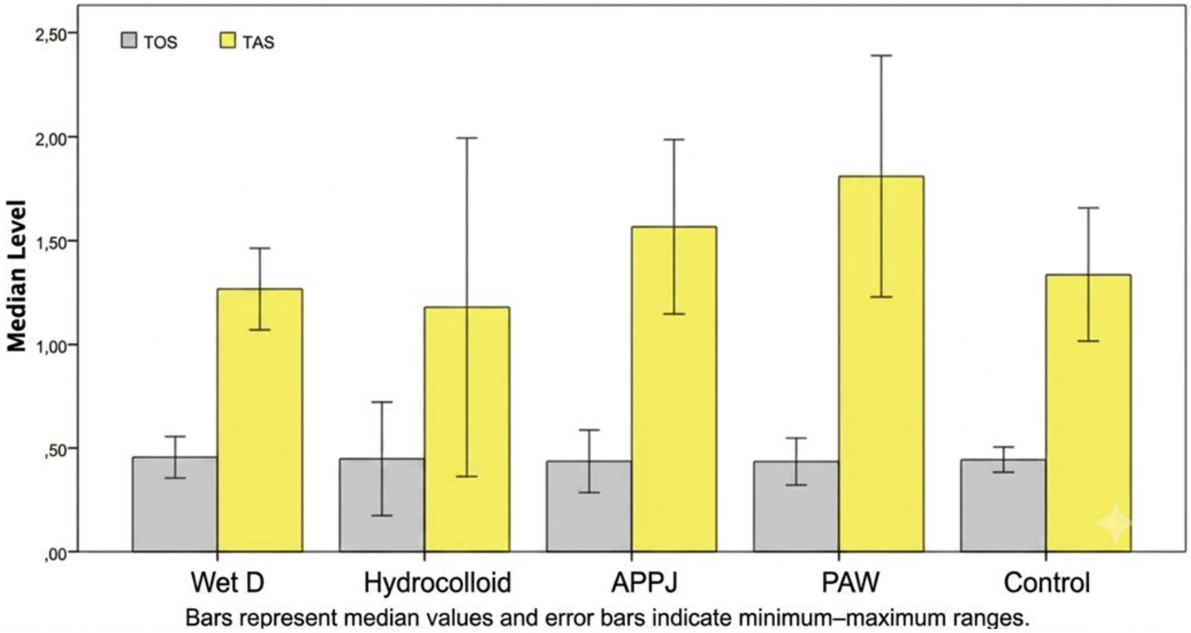

Figure S10. Median TAS and TOS levels among experimental groups. Bars represent median values and error bars indicate minimum–maximum ranges.

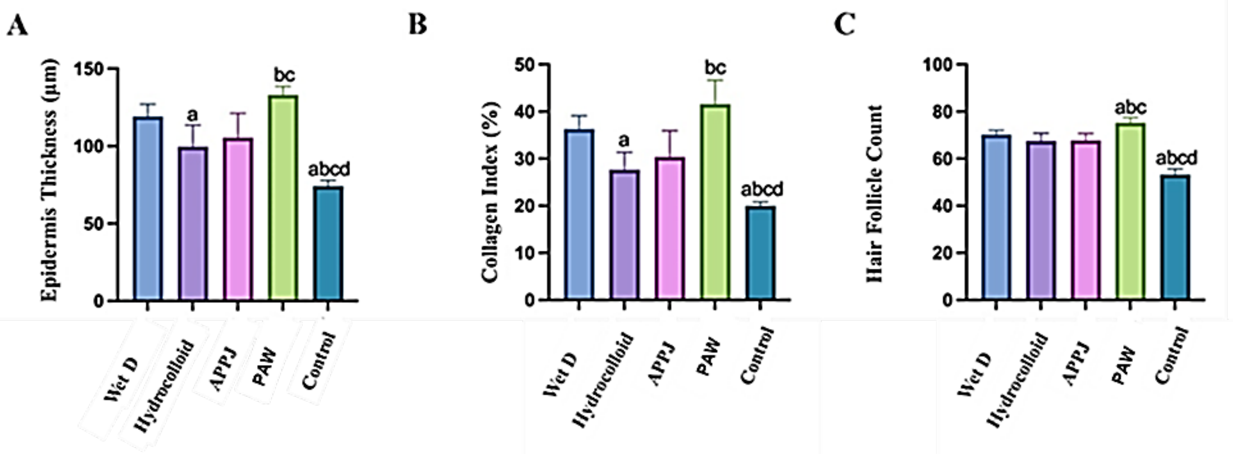

Figure S11. Quantitative analysis of histological wound healing parameters. (A) Epidermal thickness, (B) collagen index, and (C) hair follicle count. Different letters indicate statistically significant differences between groups ($p < 0.05$).

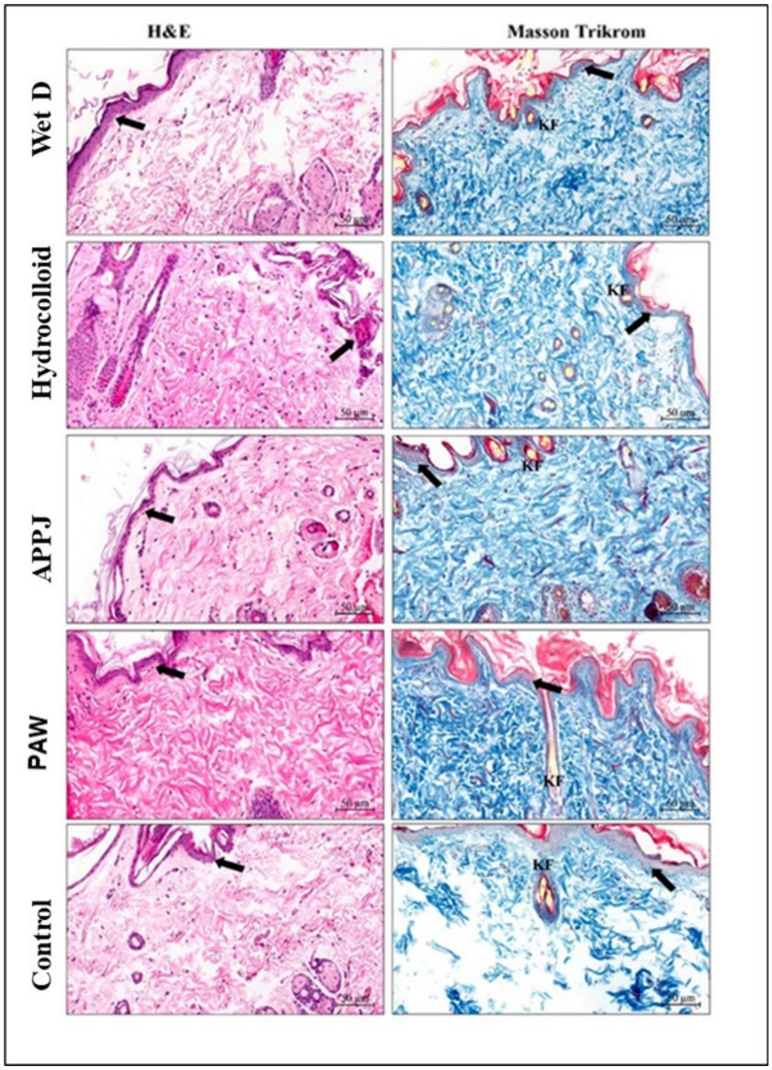

Figure S12. Hematoxylin and eosin (H&E)-stained images of skin tissue samples. The black arrow indicates epidermal thickness, and HF denotes hair follicles. Magnification: ×20; scale bar: 50 µm.
